# Performance, combustion and emission reduction characteristics of Metal-based silicon dioxide nanoparticle additives included in ternary fuel (diesel-SMME-iso butanol) on diesel engine

**DOI:** 10.1016/j.heliyon.2024.e26519

**Published:** 2024-02-16

**Authors:** Gandhi Pullagura, Joga Rao Bikkavolu, Srinivas Vadapalli, V. Varaha Siva Prasad, Kodanda Rama Rao Chebattina, Debabrata Barik, Milon Selvam Dennison

**Affiliations:** a Department of Mechanical Engineering, GITAM School of Technology, Visakhapatnam, India 530045; b Department of Mechanical Engineering, Godavari Institute of Engineering and Technology A, Rajahmundry, A.P, India 533296; c Department of Marine Engineering, Andhra University, Visakhapatnam, India 530003; d Department of Mechanical Engineering, Karpagam Academy of Higher Education, Coimbatore, India641021; e Department of Mechanical Engineering, Kampala International University, Western Campus, Kampala, Uganda 20000

**Keywords:** Silicon dioxide, Ternary fuel, Iso butanol, Performance, Combustion, Emissions

## Abstract

Biodiesel has long been recognized as a viable alternative energy source. In order to enhance the quality, and performance of biodiesel-diesel fuel blends while reducing air pollution from combustion, additives must be employed. The present research aims to focus on the addition of SiO_2_ novel nanoparticles (at a concentration of 30, 60, and 90 mg/L) in the ternary fuel (TF) blend (75% of Diesel+ 15% of Sea Mango Methyl Ester (SMME15) + 10% of iso-Butanol on a volume basis) to determine engine performance, combustion, and emission characteristics of a 1-cylinder, direct injection, liquid-cooled, diesel engine. In addition to this, a stability analysis for the prepared samples was also carried out as per the ASTM standard. From the investigation, it was observed that, when the nanoparticles mixed with ternary fuel (i.e., TFSi60), the brake thermal efficiency (BTE), In-cylinder pressure (ICP), and net heat release rate (NHRR) were improved by about 10.09, 17.4, and 10.73 % respectively. Whereas the brake-specific fuel consumption (BSFC) (19.13%) and hazardous pollutants like carbon monoxide (CO) (20.06%), unburnt hydrocarbons (UHC) (13.9%), nitrogen oxides (NOx) (11.3%), and smoke (11.2%) were significantly decreased. From the above observations, it is concluded that using a ternary fuel blend with nano additives improves engine performance and combustion while lowering toxic emissions.

## 1 Introduction

Globally, fossil fuels have been used roughly 50% in transportation and will be depleted in a decade [1,2]. Furthermore, successful usage has an impact on greenhouse gas (GHG) emissions, prompting researchers to work on alternative fuels that may be renewable and may replace conventional diesel fuel [3,4]. Biodiesel is morally permissible among numerous alternatives (vegetable oils, alcohols, etc.) since it has superior physio-chemical characteristics, is renewable, non-toxic, is low in sulfur, is eco-friendly, and has a greater oxygen content than diesel [5,6]. However, biodiesel commercialization is hindered by a lack of cold flow parameters (cloud point (CP), pour point (PP)), higher NOx emissions, poor performance, poor oxidation stability, and piston sticking issues [7,8]. These limitations prompted various approaches, including the inclusion of alcohols and nanoparticles as additives, thus increasing physio-chemical properties, performance, and combustion parameters, and reducing pollutants [9,10].

The inclusion of different additives blended in biodiesel-diesel mixtures has become very common since their ability to substitute diesel fuel [11,12]. The nanoparticles comprise higher thermal conductivity and a higher surface-to-volume ratio and support as a catalyst to improve physiochemical properties and overall performance of the engine and reduce emissions [2,13]. In contrast, iso-butanol is a promising alcohol additive compared to ethanol, methanol, and n-butanol due to its superior properties and higher energy content [14,15]. Iso-butanol is an isomer of butanol. Thus, the n-butanol and iso-butanol belong to a similar family having the same molecular weight and different chemical structures [16,17]. The long-chain iso-butanol additive can be easily blended in biodiesel-diesel blends (small-chain blends) and possess high energy content [18,19].

Many researchers focused on the influence of different additives in biodiesel-diesel mixtures. For example, Rangabashiam, D. et al. (2019) [20] investigated that silicon dioxide (SiO_2_) nano additives were included in pure diesel at a different particle sizes of 10 and 20 nm. The addition of nanoparticles reduced emissions greatly. In another study, Khatri, D and Goyal R (2020) [21] investigated the influence of SiO_2_ nano additives (at concentrations of 25, 50, 75, and 100 ppm) on water diesel emulsified fuel (WDEF) at different injection timings. The findings showed that the reduction of NOx, HC, CO, and smoke opacity was found to be reduced with a nano additive dosage of 50 ppm. In a study, Prabhu Kishore Nutakki, P. K. et al. (2021) [22] experimented on a common rail direct injection (CRDI) compression ignition (CI) engine when the engine is fuelled with mahua methyl ester included SiO_2_ nanoparticles (at a concentration of 40, 80, and 120 ppm). The performance was enhanced while the pollutants were reduced except for NOx emissions. Similarly, Wei, J et al. (2021) [23] experimented with a modern dual fuel (SiO_2_ (at a dosage of 25, 50, and 100 ppm) included in methanol) in a CI engine. The addition of SiO_2_ in methanol reduced all emissions and improved overall performance.

A few scholars explored the addition of alcohol additives in diesel and biodiesel samples [24]. In a study, Algayyim S. J. M et al. (2018) [25] investigated that the addition of n-butanol and iso-butanol mixed with diesel fuel and mentioned that the inclusion of iso-butanol (at 10% and 13% concentration) improved combustion and performance properties and a significant reduction in emissions. Similarly, n-butanol (at 10% and 13% concentrations) decreased emissions due to the energy necessary to break the C–H bonds of n-butanol being less than iso-butanol. Higher concentrations of alcohol reduced NOx emissions, and both n-butanol and iso-butanol can be blended to reduce emissions and improve performance and combustion parameters. In another study, Xiao H et al. (2020) [26] experimented with the effect of iso-butanol and biodiesel mixtures on a CI engine and concluded that the ignition delay was prolonged with iso-butanol addition and improved combustion and performance characteristics. Further, the emissions were reduced due to the iso-butanol effect. Similarly, a few researchers focused on both nano and alcohol additives. Pullagura G et al. (2022) [27] stated that the inclusion of TiO_2_ and dimethyl carbonate (DMC) blended in a biodiesel-diesel sample. Finally, the incorporation of 50 ppm of TiO_2_ nano additives and 10% of DMC in the biodiesel-diesel sample improved overall performance and decreased pollutants. In another research, Venu H et al. (2021) [28] explored the inclusion of nanoparticles in ternary fuel (diesel-biodiesel-ethanol) samples at concentrations of 10, 20, and 30 ppm in a CI engine and suggested that the inclusion of 20 ppm Al_2_O_3_ nano additives in ternary blend enhanced combustion and performance characteristics and reduced emission levels.

Some authors used different techniques to enhance the engine performance, combustion, and emission parameters. In a study, Elkelawy, M et al. (2022) [29] stated that biodiesel is prepared from waste cooking oil using a minimal concentration of alcohol in the transesterification process and fuelled in a CI engine. From test results, it was concluded that the BTE improved by 9.6% whereas, the BSFC and exhaust temperature were decreased by 16, and 7.6% for 80% of biodiesel and 20% of diesel blend. While NOx emissions are decreased for the B20 (20% of biodiesel mixed in 80% of diesel) blend. In another study, Elkelawy, M et al. (2023) [30] revealed that the heterogeneous transesterification is carried out for the preparation of biodiesel from waste cooking oil feedstock using 0.01 % mass of TiO_2_ nano-catalyst, 0.3 % mass NaoH and oil to methanol ratio of 1:10 and resulted in 95% biodiesel yield when the reaction is held for 60 °C for 60 min. The engine was fuelled with biodiesel-diesel with cadmium (II) based supramolecular nano additives and the findings are observed to be lower CO, HC, and NOx emissions for 75 ppm of nano additions. It was also found that the BTE improved to 31.2%, and EGT was observed to rise for all the blends. In another study, Elkelawy, M et al. (2018) [31] showed that the combined mechanisms (chemical kinetic oxidation and skeleton mechanism) were validated with experimental data using various combustion conditions in HCCI engines for diesel-biodiesel-ethanol blends. Finally, it was concluded that the mechanism results in data were valid at various operating conditions. In another investigation conducted by Zhang, Z et al. (2023) [32] a 3-D dimensional diesel particulate filter model is developed using AVL Fire software to find the key structural factors. The results found that the pressure drop is reduced by increasing the filter diameter, length, and wall thickness and also revealed that the filtration efficiency can be improved by decreasing CPSI/wall thickness. In another study, Zhang, Z. et al. (2023) [33] summarized the regeneration technology of diesel particulate filters, filter structure, new catalyst formula, accurate soot prediction, safe and reliable regeneration strategy, uncontrolled regeneration, controlling methods, and additional emissions. A few articles concentrated on alcohol addition to enhance the performance and reduce emissions for example, Donghui Qi et al. (2022) [34] investigated the influence of a two-stage injection system (pilot injection timing, and pilot injection quantity) using diesel-palm oil ethanol micro-emulsions on common rail injection (CRDI) engine. The results show that the CP and HRR are higher for D60P30E10 lend at a higher load. It was also noticed that the CP and HRR are improved marginally with pilot injection timing whereas, increasing pilot injection quantity reduced HRR first and then increased. It was concluded that the NOx and Particulate matter was reduced for microemulsion fuel using a combined strategy. In another study, D. H. Qi et al. (2021) [35] stated the effect of EGR (10, and 20 % rate) when diesel, palm oil, and ethanol are used in CI engines. The findings revealed that the BSFC is improved and no change in BTE. Whereas, the ignition delay (ID) was found to be short for D60P30E10 and D50P40E10 blends. The CP is higher for D60P30E10 and HRR, and NOx was the same for all test fuel blends. But, NOx emissions are lower for higher EGR rates. It was also concluded that the variation of particle number concentration reduces the NOx and soot emissions for D50P40E10 and D40P30E10 blends with a 20% EGR double injection strategy.

The present research is aimed to focus on the addition of SiO_2_ novel nanoparticles (at a concentration of 30, 60, and 90 mg/L) in the ternary fuel (TF) blend (75% of Diesel+ 15% of Sea Mango Methyl Ester (SMME15) + 10% of iso-Butanol on a volume basis) to determine engine performance, combustion, and emission characteristics of a 1-cylinder, direct injection, liquid-cooled, diesel engine. The SMME was prepared from sea mango oil and various other fuel blends. The physicochemical properties of prepared blends were tested to check their stability and within the permissible range, and it was observed that all the prepared blends are within the ASTM standards. TF blend was prepared by blending 75% of Diesel, 15% of SMME, and 10% of iso-Butanol on a volume basis. The silicon dioxide nanoparticles included at a dosage of 30, 60, and 90 mg/L in TF blends are found to be stable after 30 days which was confirmed by characterization such as SEM.

### 1.1 The novelty of the present work

**Fig. 1:**
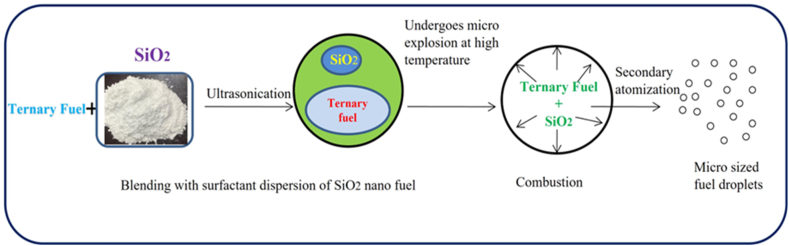
Illustration of secondary atomization effect for Ternary fuel (75% of Diesel+ 15% of Sea Mango Methyl Ester (SMME15) + 10% of iso-Butanol) SiO_2_ nano fuel samples.

To date, the alcohol additives/nano additives were added in biodiesel-diesel fuel samples to improve performance, combustion, and emission characteristics, but there was an issue with NOx emissions and cold flow properties. Very less literature study was noticed with nanoparticles and alcohol as additives added in the biodiesel-diesel blend. The addition of SiO_2_ in combination with ternary fuel (TF) (diesel (75% vol) – Sea mango methyl ester (SMME) (15% vol) – iso-butanol alcohol (10% vol)) blend is not yet explored in any investigation. Hence, the current investigation is aimed to focus on the addition of SiO_2_ nanoparticles (at a concentration of 30, 60, and 90 mg/L) in the TF blend to determine engine performance, combustion, and emission characteristics. In addition to this, the spray jet momentum of the fuel was also improved due to the extreme secondary atomization of the blend. And it is evident in Fig. 1. Additionally, the transmittance of TF-incorporated nanoparticles was analyzed using a spectrophotometer to ensure complete combustion, the stability of nanoparticles at different dosages in the TF sample.

## 2 Materials & methodology

### 2.1 Materials

In the current study, biodiesel is prepared from sea mango. The scientific name of this fruit is Cerbera odollam, also called poisonous fruit (toxic), and appears in thick green, having a diameter of 3–5 cm. It is mostly available in Africa, Asia, and Australia. The sea mango belongs to the Apocynaceae family and is considered a feedstock for biodiesel production because it possesses 40–50% of oil content and is obtained from Arakuveli, Vishakhapatnam district, India [36]. The required solvents, such as n-hexane (90%), methanol (99.9%), and KOH pallets (85%) obtained from Molekula Biokemix Limited, Hyderabad, India, and used directly without any further treatment.

### 2.2 Extraction of the oil

Initially, the seeds were dried in an oven at 50 °C until the moisture content was decreased. The raw oil was extracted using the Soxhlet equipment and n-hexane solvent after the seeds were ground into fine particles and added to the apparatus. Finally, the oil was extracted using a rotary evaporator. The fatty acid concentration of extracted oil was then found to be 6.2, allowing for both pre-esterification and transesterification. Table 1 presents the composition of free fatty acids for raw oil.

**Table 1:** Free fatty acid composition in the oil.

Free fatty acid composition	% Composition
Palmitic acid [C16:0]	31.50
Stearic acid [C18:0]	3.62
Oleic acid [C18:1]	47.28
Linoleic acid [C18:2]	16.62
Acid value (mg KOH/g)	12.84
Alpha-Linolenic acid	1.2
Free fatty acid (%)	6.2
Water content (%)	0.92

### 2.3 Preparation of biodiesel

**Fig. 2:**
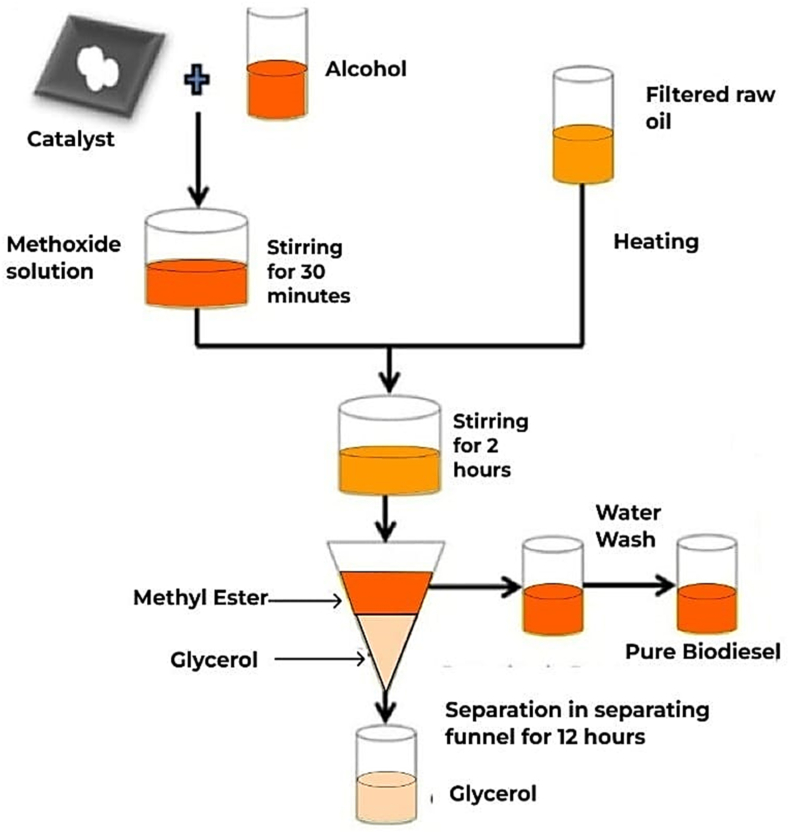
Represents the preparation of biodiesel.

In the pre-esterification process, the weighed oil sample and methanol were collected in a round neck flask at a molar ratio of 1:6 including 1.0 wt% of KOH, and heated continuously for 60 min using a heating mantle which is maintained at a temperature of 65 °C and at a stirrer speed of 200 rpm. 4 wt% KOH was used as a catalyst in the pre-esterification process [37]. The same procedure is carried out in the transesterification process for 2 h at a molar ratio of 1:6, at a temperature of 50 °C, and at a stirrer speed of 200 rpm. Following this, the solution was left to reach a steady condition for 24 h using a funnel. After this period, the solution was discovered to be two layers. The bottom layer was revealed to be murky and removed, while the upper layer was separated. The solution was then rinsed and dried numerous times [10]. After transesterification, the fatty acid value was determined to be 1.2, and the maximum yield of transesterification was found to be 92% using Eq (1). below. Table 2 shows the physio-chemical parameters of the prepared biodiesel and other samples. Fig. 2 illustrated the experimental steps involved in biodiesel production.

(1) $$\text{Yield}\;\%=\frac{\text{Total}\;\text{weight}\;\text{of}\;\text{methyl}\;\text{esters}}{\text{Total}\;\text{weight}\;\text{of}\;\text{oil}\;\text{in}\;\text{the}\;\text{sample}}\times 100\;\%$$

**Table 2:** Physiochemical Properties of neat Diesel, biodiesel, TF, and TF-SiO_2_ samples (at different concentrations).

Fuels/Blends properties	Testing method	Diesel Limit		Biodiesel Limit		SMME15	TF	TFSiO_2_30	TFSiO_2_60	TFSiO_2_90
		ASTM D975	Petro-diesel	ASTMD6751	EN14214					
Density (kg/m^3^ at 15 °C)	ASTM D1298	850	835	880	860 to 900	870	834	839	845	848
Kinematic viscosity (mm^2^/s at 40 °C)	ASTM D445	2.0 to 4.5	2.80	1.9 to 6.0	3.5 to 5.0	3.86	3.6	3.65	3.73	3.75
Calorific value (Mj/kg)	ASTM D2015	42 to 46	44.5	–	35	42.4	40.80	41.42	42.18	42.60
Cetane Index	ASTM D976	≥47	52	47	–	50	52	56	59	60
Flashpoint (°C)	ASTM D93	60 to 80	69	100 to 170	120 min.	120	84	88	95	92
Fire point (°C)	ASTM D93	–	75	–	–	134	89	93	100	100
Cloud point (°C)	ASTM D2500	−35 to +15	2	−3 to −12	–	3	1	−1	−2	−2
Pour point (°C)	ASTM D97	−15 to +5	1	−15 to −16	–	−4	−4	−6	−10	−8

### 2.4 Preparation of nano fuel blends

**Fig. 3:**
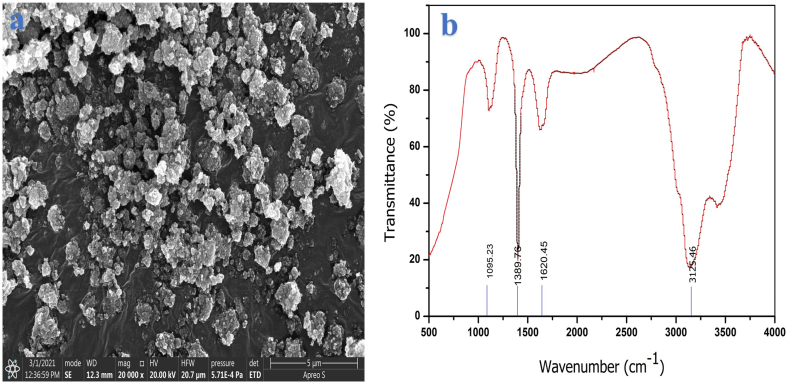
Fig. 3(a). SEM image for the SiO_2_ nanoparticles and 3(b) FTIR of SiO_2_ nanoparticles.

**Fig. 4:**
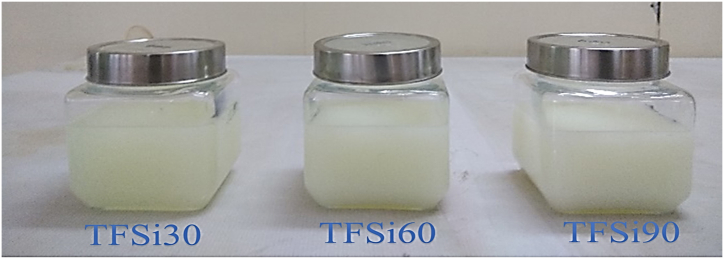
Represents diesel-TF blends with different dosages of nanofluids.

SiO_2_ nanoparticle's surface was modified using QPAN 80 surfactant and was blended at a ratio of 1:1 with solvent. The blend was sonicated for 15 min and allowed solvent to evaporate. A scanning electron microscope (SEM) is used to investigate the morphology of SiO_2_ nanoparticles. Fig. 3(a) depicts the SEM image findings achieved for SiO_2_. At 20,000X magnification, the SEM image demonstrates that SiO_2_ nanoparticles are spherical in form with a smooth surface and an average particle size of 35 nm. Fig. 3(b) shows an FTIR image of SiO_2_ nanoparticles which shows absorption peaks at 1095.23 and 1389.76 cm^−1^ which is owing to silicon-oxygen bond vibration thereby signifying the product formation. Since nanoparticles are smaller in size than the diameter of a fuel injector nozzle, they will not hinder fuel flow in the nozzle. The first sample includes 85% vol of diesel blended with 15% vol of SMME and is referred to as SMME15. The second sample contains commercial diesel of 75% vol +15% vol of SMME +10% vol of Iso-butanol, and this blend is referred to be Ternary fuel (TF). The surface-modified SiO_2_ was blended in TF at 30, 60, and 90 mg/L concentrations using a mechanical disseminator and ultrasonic pulsing frequency technique (Hielscher ultrasonic, 160 W, 40 kHz, 30 min for each sample) to eliminate aggregation and provide better stability. These samples were termed TFSi30, TFSi60, and TFSi90, respectively. The prepared samples are observed for 30 days for stability and found to be uniform and homogeneous. Fig. 4 shows various samples of TF with various concentrations of nanofluid blends. The physio-chemical properties are determined per ASTM standards for diesel and prepared samples.

### 2.5 Thermal conductivity

**Fig. 5:**
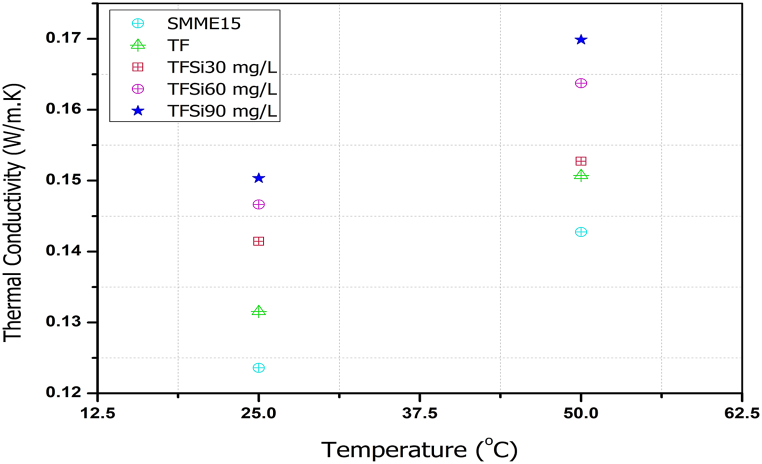
Thermal conductivity of the various fuel blends with different temperatures.

The thermal conductivity (Model: TRI-4, Trident) of the various fuel blends vs temperature is illustrated in Fig. 5. The results show that an increase in thermal conductivity was observed for all fuel samples except the B20 blend, which can be explained by various mechanisms such as Brownian motion and clustering/interfacial layer. In general, nano fuels with mixed surfactants have higher thermal conductivity. Thermal conductivity is higher for the SiO_2_ blended TF samples than the TF blend. This could be mainly due to the higher stability and transport mechanism and, higher thermal & electrical conductivity, and heat transfer rate of SiO_2_ [38]. Similar results are observed in other investigations [27,39].

### 2.6 Stability test analysis

**Fig. 6:**
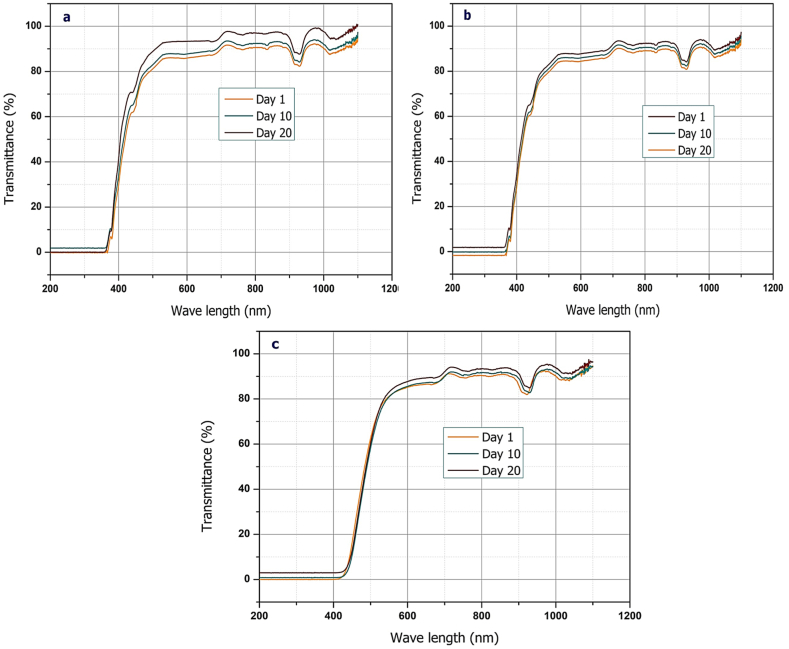
Transmittance vs. wavelength (stability test): (a) TFSi30, (b) TFSi60, and (c) TFSi90.

To ensure complete combustion, the stability of nanoparticles at different dosages in the TF sample is crucial. Under these situations, dispersion becomes significant because it prevents the separation, agglomeration, and sedimentation of nanoparticles. In the present work, the transmittance of TF-incorporated nanoparticles was analyzed using a spectrophotometer. The test was conducted for 30 days, with readings taken every 10 days on Days 1–20. Fig. 6(a)–(c) shows how transmittance changes as a function of wavelength. As can be seen, the transmittance range was investigated using the same methodology for all test fuel blends. The transmittance range was marginally improved on Days 10 and 20 correlated to Day 1 regardless of test fuel. The drastically decreased transmittance suggests that nanoplatelets in the TF have exhibited good stability over a 20-day period. The opaque nanoparticles in the fuel sample prevent any light from passing through it. Hence, the fuel mixtures demonstrate stronger light absorption (lower light transmittance), indicating superior stability.

### 2.7 Engine setup and uncertainty

**Fig. 7:**
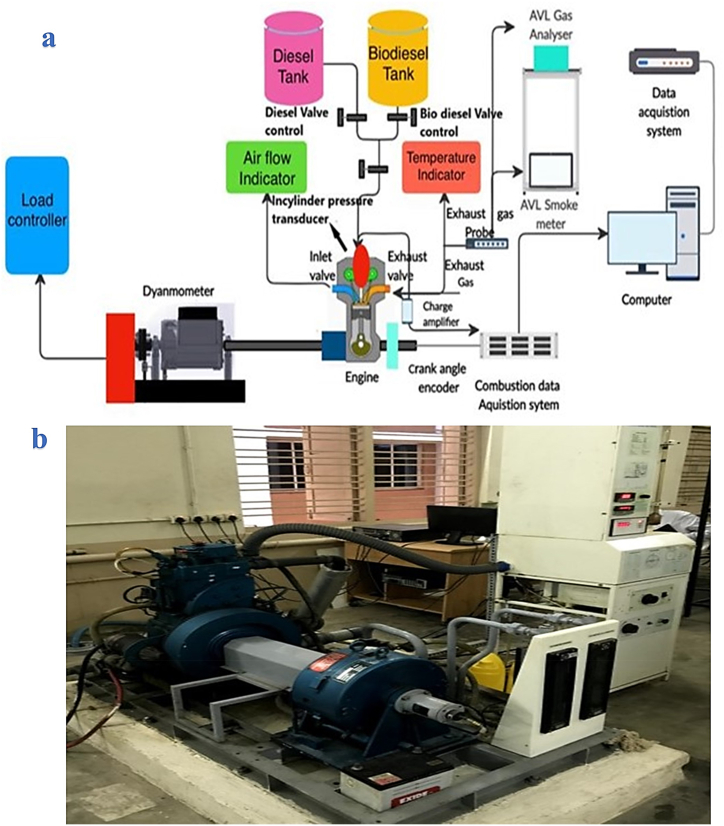
Experimental Setup (a) Schematic diagram (b) Photograph of the setup.

In the current experimental work, a 1-cylinder, 4-stroke, Kirloskar model of power 5.2 kW, speed of 1500 rpm, eddy current load bank, and the water-cooled engine was used along with AVL 5-Gas analyzer set up. The schematic diagram and the photograph of the experimental setup are shown in Fig. 7 (a, b). The engine details are given in Table 3. A digital converter is coupled with all required sensors and verified to reduce the errors then it is connected to the computer. The pre-installed engine soft analyses the data for 100 cycles. A detailed description of the experimental setup and tools used for the data collection is already documented in the author's earlier published articles [10,12,13,37].

**Table 3:** Specification of the CI engine.

S·NO.	Engine parameters	Specifications
1	Engine model	TAF-1, Kirloskar
2	No. of cylinders/No. of strokes	1/4
3	Bore diameter/Stroke length	100/105 mm
4	Rated power	5.2 Kw
5	Rated speed	1500 rpm
6	Compression ratio	17.5
7	Gas analyzer and smoke meter	AVL make
8	Ignition timing	23^0^ bTDC
9	Type of Ignition	Compression-Ignition
10	Dynamometer	Eddy Current Type
11	Fuel injection	Direct injection

The net heat release rate is determined using the first law of thermodynamics stated in equation (2). The CI engine was started with standard diesel fuel for 30 min, and the test results were noted for base reference. Then the tested samples (SMME15, TF, TFSi30, TFSi60, TFSi90) were used to examine performance, combustion, and emissions parameters. These tests were done several times, and the mean values were reported. The engine setup is illustrated in Fig. 7. The total uncertainty of experiments equation (3) is found to be 2.22% and shown in Table 4.

(2) $$\frac{{dQ}_{total}}{d\theta }=\left(\frac{\gamma_{sp.heat}}{\gamma_{sp.heat}-1}\right)\left(P_{cyl}\right)\left(\frac{dV}{d\theta }\right)+\left(\frac{1}{\gamma_{sp.heat}-1}\;\right)\left(V\right)\left(\frac{dP}{d\theta }\right)+\left(\frac{dQ_{w}}{d\theta }\right)$$

Where: = $\frac{c_{p}}{c_{v}}$.

**Table 4:** Accuracies and the uncertainties used in the evaluated parameters.

Sl. No.	Instrument/Device	Range	Accuracy	Uncertainty
1	Load indicator	0.25–5 kW	±10 W	±0.2
2	Exhaust gas temperature	0–1300 K	±1 ^O^C	±0.5
3	Exhaust gas analyzer	CO (0–9.99 %)	±0.06	±0.01 % Vol.
		HC (0–15000 ppm)	±12	±1 ppm
		NO_X_ (0–5000 ppm)	±12	±0.5 ppm
4	Speed	–	±1 rpm	±1
5	EGT	–	±1 °C	±0.15
6	Load indicator	250–5000 W	±10 W	±0.2
7	Thermocouples	0–1300 K	±1 °C	±0.15
8	Pressure transducer	0–250 bar	±1	±2
9	Burette (Fuel Consumption)	1–30 cm^3^	±0.2	±0.5

The following equation illustrates the overall uncertainty:

(3) $$=\sqrt{{u_{(Tech}}^{2}+{\left(u_{sfc}\right)}^{2}+{\left(u_{CO}\right)}^{2}+{\left(u_{HC}\right)}^{2}+{\left(u_{NOx}\right)}^{2}+{\left(u_{BP}\right)}^{2}+{\left(u_{EGT}\right)}^{2}+{\left(u_{N}\right)}^{2}\;}$$

$$=\sqrt{{\left(1.5\right)}^{2}+{\left(0.4\right)}^{2}+{\left(0.01\right)}^{2}+{\left(1\right)}^{2}+{\left(0.5\right)}^{2}+{\left(0.2\right)}^{2}+{\left(0.5\right)}^{2}+{\left(1\right)}^{2}\;}=\pm \;2.22$$

where: u_Tech_: Uncertainty of Brake Thermal Efficiency.

u_sfc:_ Uncertainty of Specific fuel consumption.

u_CO_: Uncertainty of CO emission.

u_HC_: Uncertainty of HC emission.

u_NOx_: Uncertainty of NOx emission.

u_BP_: Uncertainty of Brake power.

u_EGT_: Uncertainty of Exhaust gas temperature.

u_N_: Uncertainty of Engine speed.

The formulas used for calculating the performance and emission data are given below.

Brake thermal efficiency (%)

(4) $$\eta B_{th}=\frac{BP}{m_{f}\times CV}$$

The emission values for CO in %, HC in ppm, and NO_x_ in ppm can be converted into g/kWh by using Equations (5), (6), (7) respectively.

Brake-specific CO emissions in (g/kWh)

(5) $$\frac{\left(m_{a}+m_{f}\right)\;X\;\left(1000\;X\;3600\right)\;X\;\left(Molecular\;weight\;of\;CO\right)\;X\;\left(CO\;in\;\%\right)}{\left(Molecular\;weight\;of\;air\right)\;X\;\left(Brake\;power\left(kw\right)\right)\;X\;100}$$

Brake-specific HC emissions in (g/kWh)

(6) $$\frac{\left(m_{a}+m_{f}\right)\;X\;\left(1000\;X\;3600\right)\;X\;\left(Molecular\;weight\;of\;HC\right)\;X\;\left(HC\;in\;\%\right)}{\left(Molecular\;weight\;of\;air\right)\;X\;\left(Brake\;power\left(kw\right)\right)\;X\;100}$$

Brake specific NOx emissions in (g/kWh)

(7) $$\frac{\left(m_{a}+m_{f}\right)\;X\;\left(1000\;X\;3600\right)\;X\;\left(Molecular\;weight\;of\;NOx\right)\;X\;\left(NOx\;in\;\%\right)}{\left(Molecular\;weight\;of\;air\right)\;X\;\left(Brake\;power\left(kw\right)\right)\;X\;100}$$

## 3 Results and discussions

### 3.1 Engine performance characteristics

#### 3.1.1 Brake thermal efficiency

**Fig. 8:**
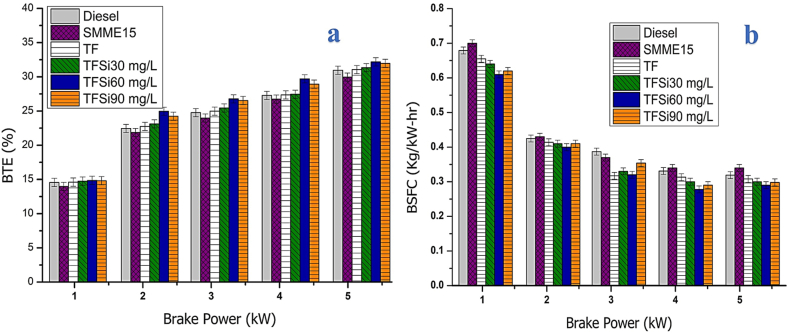
Fig. 8(a). Illustrates the effect of BTE vs BP. Fig. 8(b) Variation of BSFC vs BP for various fuels.

BTE was observed to be improved with increasing Brake Power (BP) for various fuels, as illustrated in Fig. 8(a). The BTE was calculated as per the parameters given in Equation (4). The BTE of nano additives blended TF fuel (TFSi60) was higher when correlated to other nano ternary blends, SMME15, and diesel which is attributed to catalytic activity refinement for micro-explosion and better combustion characteristics [40,41]. In addition to this, the evaporation and atomization of nano TF blends were much improved due to higher heat transfer rates which causes reduced ignition delay and enhanced BTE [2,19]. The BTE of the TF blend was much better than diesel and SMME15 samples which is attributed to the oxygen content in the iso-butanol and SMME. The lowest BTE was observed for the SMME15 blend, owing to higher viscosity and lower calorific value. The BTE was found to be 31.2%, 29.82%, 31.86%, 32.24%, 34.35%, and 32.89% for Diesel, SMME15, TF, TFSi30, TFSi60, and TFSi90 blends at higher BP. Maximum BTE was enhanced by 10.09% for the TFSi60 blend correlated to diesel at maximum BP [40,42].

#### 3.1.2 Brake-specific fuel consumption

Fig. 8(b). Represents the effect of BSFC for standard diesel, SMME15, TF, and TF blended with SiO_2_ nanoparticles with reference to the engine power. The blend SMME15 contains higher viscosity and less calorific value due to which atomization and vaporization properties are poor to produce the same output and it consumes more fuel. At the same time, the ternary fuel blend reduced fuel consumption due to the addition of iso-butane. Oxygen content and improved fuel properties cause effective combustion and reduced fuel consumption than diesel. The inclusion of SiO_2_ nanoparticles in the TF blends enhanced the combustion process. The nanoparticles addition in the TF blend provides abundant oxygen to burn injected fuel completely and improved physiochemical properties for reducing fuel consumption [43]. In contrast, the higher dosage of nano additives in the TF blend improves viscosity and performs similarly to the SMME15 blend, thus improving fuel consumption. The BSFC is found to be 0.328, 0.345, 0.312, 0.296, 0.279, and 0.284 kg/kW hr for diesel, SMME15, TF, TFSi30, TFSi60, and TFSi90 blends at maximum BP. The decrement in BSFC is observed to be 19.13% for the TFSi60 sample. The reduced BSFC is owing to a greater cetane number, improved spray pattern, and better atomization characteristics [40].

### 3.2 Combustion characteristics

#### 3.2.1 In-cylinder pressure

**Fig. 9:**
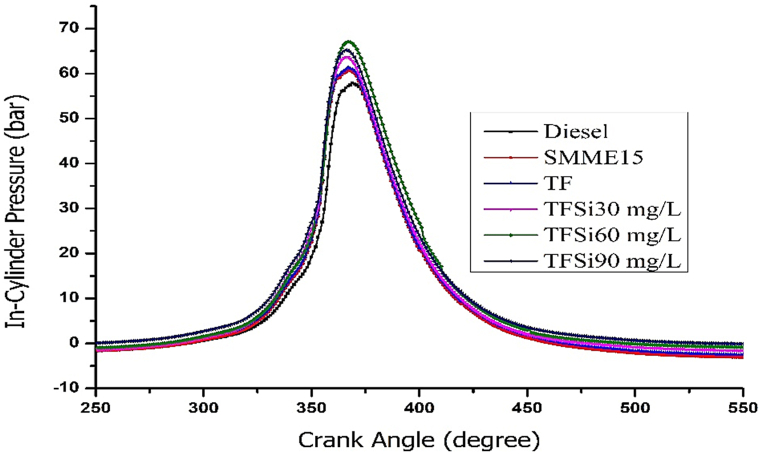
Shows the variation of In-cylinder pressure vs Crank angle for prepared samples.

Fig. 9. The influence of in-cylinder pressure (ICP) on the crank angle for tested samples is depicted. It is found from the figure that the lower pressure was noticed for diesel at 57.6 bar. Whereas the peak pressure of ICP was noticed for TFSi60 mg/l blend at 67.23 bar, which is attributed to improved cetane number and lower viscosity of the tested sample. Further, the atomization properties of ternary fuel were further improved with improved physio-chemical properties. The ICP for diesel, SMME15, TF, TFSi30, TFSi60, and TFSi90 were noticed to be 57.6 bar, 62.72 bar, 63.36 bar, 64.73 bar, 67.23 bar, and 65.65 bar, respectively. The ICP is improved to 17.4% for the TFSi60 mg/l blend compared to diesel. Nevertheless, the presence of iso-butanol and B15 blend possesses higher oxygen content which aids in achieving complete combustion [21,40].

#### 3.2.2 Heat release rate

**Fig. 10:**
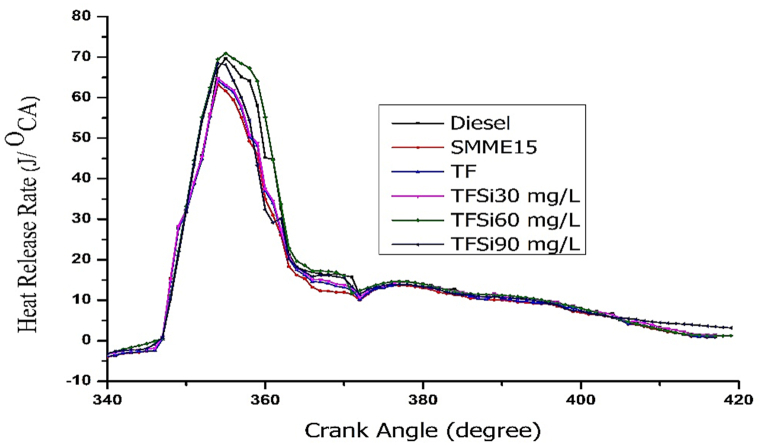
Illustrates Heat Release Rate vs Crank angle for tested fuels.

Fig. 10. Represents the variation of HRR vs crank angle for different fuel blends. It is evident from the figure that the HRR of diesel is found to be lower than all the fuel samples, which is owing to the accumulation of fuel samples in the premixed zone, lower cetane number, and major fuel characteristics. While the HRR of diesel, SMME15, TF and nano included TF blends (TFSi30, TFSi60, and TFSi90) are observed to be 65.23J/°CA, 65.75 J/°CA, 66.56 J/°CA, 70.89 J/°CA, 72.23 J/°CA, and 71.76 J/°CA respectively. The improved HRR was found to be 10.7% for the TFSi60 blend than diesel fuel. The existence of iso-butanol and a lower amount of biodiesel in the TF blend reduced accumulation in the primary combustion zone and shortened ignition delay. Which means the physical delay is improved due to the addition of iso-butanol because iso-butanol possess higher evaporation characteristics. Thus, causing to complete physical delay (compressibility factor of the fuel). Once physical delay is completed thus facilitating quick chemical reactions and causing shorter chemical delay. Thus, exhibiting higher HRR. Nevertheless, the addition of nanoparticles facilitates the initiation of combustion in the primary stage of combustion, which is owing to improved surface area and heat transfer rate [23,40].

### 3.3 Emission characteristics

#### 3.3.1 Carbon monoxide

**Fig. 11:**
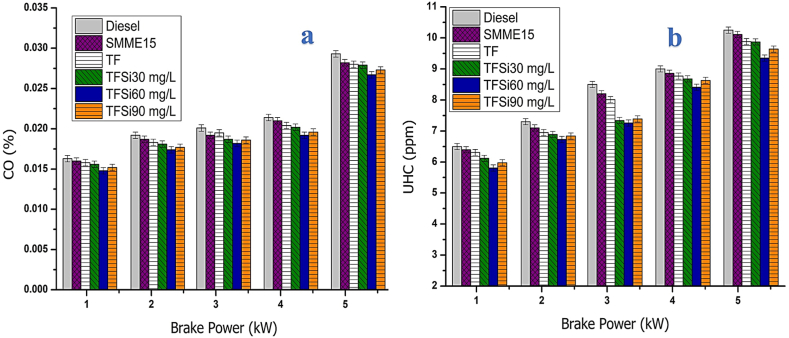
Fig. 11(a). Illustrates the effect of CO vs BP. Fig. 11(b). Effect of HC vs BP for various fuels.

The effect of CO emissions with BP for different fuel samples is illustrated in Fig. 11(a). The CO values It is noticed from the figure that the CO emissions are reduced for all fuel blends except diesel since all the blends are rich in oxygen content due to the addition of iso-butanol and SMME. The addition of iso-butanol in the TF blend prevents fuel-rich zones and acts as a combustion enhancer due to atomization characteristics [40]. In addition, the high reactivity of SiO_2_ nanoparticles inclusion reduces ignition delay and allows for rapid combustion inside the cylinder, which is owing to the high surface-to-volume ratio [44]. The CO emissions were found to be 28.9, 27.21, 26.78, 25.82, 22.34, and 23.65% for diesel, SMME15, TF, TFSi30, TFSi60, and TFSi90 fuel samples at higher BP. The maximum reduction of CO emissions is 20.6% compared to diesel fuel.

#### 3.3.2 Hydrocarbon emissions

The effect of HC emissions against BP for different fuel samples is depicted in Fig. 11(b). The HC emissions from the engine represent lower performance, and HC is found to be high for neat diesel compared to all fuel samples. The HC emissions emitted for diesel, SMME15, TF, TFSi30, TFSi60, and TFSi90 blends were 10.22, 9.96, 9.87, 9.62, 8.79, and 9.42 ppm, respectively, at higher BP. Pure diesel possesses higher HC emissions which are attributed to wall films in cold quench zones when a rich air-fuel mixture is supplied. The addition of iso-butanol in the SMME15 blend speeds up the evaporation rate of the fuel droplets due to the low boiling point of the isomer/iso-butanol, which leads to reduced ignition delay period and rapid combustion, thus, lowering HC emissions [25]. Further, the inclusion of SiO_2_ nano additives shortens the ignition delay owing to high catalytic activity and higher surface-to-volume ratio, which causes better air-fuel mixing [40,44].

#### 3.3.3 Nitrogen oxides

**Fig. 12:**
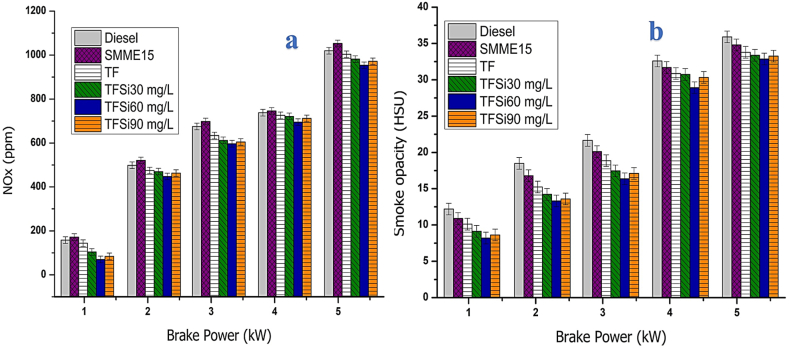
Fig. 12(a). Illustrates NOx vs BP. Fig. 12(b). Illustrates Smoke opacity vs BP.

The effect of NOx emissions for diesel, SMME15, TF, TFSi30, TFSi60, and TFSi90 blends at different BP are shown in Fig. 12(a). The major factors influencing the production of NOx are response time, cylinder mean temperature, engine design, fuel characteristics, and surrounding temperature. The NOx emissions were lower for diesel than SMME15 blend, which is attributed to more oxygen content in SMME thus causing better combustion and raising cylinder temperature [40]. The NOx emissions are improved by 6.9 % for SMME15 than diesel at a higher BP. The TF blend presents lower NOx emissions than diesel because the addition of iso-butanol improves heat of evaporation, and its lower calorific value induces the cooling effect in the combustion chamber and decreases NOx emissions [45]. Further, the NOx emissions were reduced due to the addition of SiO_2_ nano additives. The higher thermal conductivity of SiO_2_ nano additives enables them to act as a heat sink and decrease the combustion product's temperature and resulting in lower NOx [46,47]. The NOx emissions were found to be 1010, 1080, 980, 970, 957, and 966 ppm for diesel, SMME15, TF, TFSi30, TFSi60, and TFSi90 tested samples. The greatest reduction in NOx emissions was found for TFSi60 by 11.3 %.

#### 3.3.4 Smoke opacity

Fig. 12(b). Illustrates the influence of smoke opacity with BP for different fuel blends. Diesel fuel emits greater smoke emission compared to all the blends, which is attributed to the accumulation of more amount of fuel in the combustion chamber, low oxygen content in the rich fuel zones, and poor vaporization characteristics, thus forming smoke. Nevertheless, the smoke emission is produced due to incomplete/partial combustion of fuel [40]. The figure implies low smoke emission from SMME15 and TF blend as they possess higher oxygen content and rapid oxidation process in all rich fuel zones in the combustion chamber. Further, smoke emissions were found to be decreased for TF blend included with SiO_2_ nano additives which are owing to higher surface activity, greater surface area to volume ratio, better ignition properties, shorter delay period, and better evaporation rate in the presence of oxygenated blends [48,49]. TFSi90 shows a marginal improvement in smoke emissions which is owing to the higher viscosity and density of the fuel blend. The smoke opacity is reduced by 36.18, 35.12, 34.56, 33.24, 32.12, and 33.16 % for diesel, SMME15, TF, TFSi30, TFSi60, and TFSi90 samples, respectively. The highest reduction in smoke emissions was observed to be 11.2% for the TFSi60 blend correlated to diesel fuel at a higher BP.

## 4 Conclusions

The influence of SiO_2_ nano additives in ternary fuel blends on the performance, combustion, and emission parameters has been evaluated and the important finding of this investigation are listed below.

❖ Nano additives of SiO_2_ added in ternary fuel blend at 20 days gave a marginal increase in stability with reduction in the transmittance. This suggests that nanoplatelets in the TF have exhibited good stability over a 20-day period compared to day 1.
❖ Nano additives of SiO_2_ added in ternary fuel blend gave smooth operation of the engine without any abnormalities in ignition, combustion and emission.
❖ The positive impact of nano additives added in ternary fuel blend (TFSi60), confirmed the lower BSFC by 19.15% and toxic emissions (CO, UHC, NOx, and Smoke opacity by 20.6%, 13.9%, 11.3%, and 11.2% respectively when compared to neat diesel at higher BP.
❖ Similarly, TFSi60 improved BTE, ICP, and HRR by 10.09, 17.4, and 10.7%, respectively which are owing to the high surface area to volume ratio, greater catalytic action of nano additives and better atomization properties of iso-butanol in the ternary fuel blend.
❖ The effect of oxygen content in biodiesel blend and iso-butanol and catalytic activity of nano fuel blends shortened ignition delay and improved combustion rate. Thus, it is concluded that the SiO_2_ nano additive including ternary blends enhanced the overall performance of the engine up to a certain limited concentration (60 mg/l) with no engine modifications.

The adoption of the proposed technology will enhance the performance of engines as well as can led to partial saving of the mineral based fuels for the future generation. Most preferably the idea is more suitable for operating agricultural water pumps, grain crushers, small scale industrial machineries and many mores where speed fluctuations is a constraint.

**Future Scope:** This research may be expanded by using various kinds of higher alcohols with carbon allotrope nano additions in biodiesel-diesel blends with variable injection timing/injection pressures to enhance the engine performance and emission reduction attributes.

## Funding statement

No financial support was received from any institution or organization for this study.

## Declarations

The present study work was not conducted on human or experimental animals where national or international guidelines are used for the protection of human subjects and animal welfare.

## Data availability statement

The data is available in the manuscript.

## CRediT authorship contribution statement

**Gandhi Pullagura:** Conceptualization, Data curation, Formal analysis, Investigation, Methodology, Validation, Writing – original draft, Writing – review & editing. **Joga Rao Bikkavolu:** Conceptualization, Data curation, Formal analysis, Investigation, Methodology, Writing – original draft, Writing – review & editing. **Srinivas Vadapalli:** Conceptualization, Data curation, Formal analysis, Funding acquisition, Investigation, Methodology, Supervision, Writing – original draft, Writing – review & editing. **V. Varaha Siva Prasad:** Conceptualization, Data curation, Formal analysis, Funding acquisition, Investigation, Methodology, Resources, Supervision, Writing – original draft, Writing – review & editing. **Kodanda Rama Rao Chebattina:** Conceptualization, Data curation, Investigation, Methodology, Writing – original draft, Writing – review & editing. **Debabrata Barik:** Conceptualization, Data curation, Formal analysis, Funding acquisition, Investigation, Methodology, Resources, Supervision, Validation, Visualization, Writing – original draft, Writing – review & editing. **Milon Selvam Dennison:** Conceptualization, Formal analysis, Funding acquisition, Resources, Supervision, Writing – original draft, Writing – review & editing.

## Declaration of competing interest

The authors declare that they have no known competing financial interests or personal relationships that could have appeared to influence the work reported in this paper.

## Nomenclature

ASTM: American Standard for Testing Method
BSFC: Brake-Specific Fuel Consumption
BTE: Brake Thermal Efficiency
° bTDC: Degree before top dead center
° C: Degree Celsius
° CA: Degree crank angles
CI: Compression Ignition
CP: Cloud Point
CRDI: Common Rail Direct Injection
DMC: Dimethyl Carbonate
g/kWh: Gram per kilowatt hour
HSU: Hartridge Smoke Unit
ICP: In-Cylinder Pressure
J/deg CA: Joule per degree crank angle
kg/kWh: Kilogram per kilowatt hour
Kg/m^3^: Kilogram per cubic meter
kHz: Kilohertz
KOH: Potassium hydroxide
kW: Kilowatt
mg/kg: Milligram per kilogram
mg/L: Milligram per liter
MJ/kg: Mega Joules per kilogram
mm: Millimeter
mm^2^/s: Milli meter square per second
nm: Nanometer
NHRR: Net Heat Release Rate
mg KoH/g: Milligram KoH per gram
NOx: Nitrogen Oxide
PP: Pour Point
ppm: Parts per million
Rpm: Revolution per minute
SEM: Scanning Electron Microscopy
SiO_2_: Silicon Dioxide
SMME: Sea Mango Methyl Ester
SMME15: (Diesel (85% vol) – SMME (15% vol)
TF: Ternary Fuel(Diesel (75% vol) – SMME (15% vol) – iso-butanol alcohol (10% vol))
TFSi30: Surface-modified SiO_2_ was blended in TF at a dosage of 30 mg/L
TFSi60: Surface-modified SiO_2_ was blended in TF at a dosage of 60 mg/L
TFSi90: Surface-modified SiO_2_ was blended in TF at a dosage of 90 mg/L
TiO_2_: Titanium Dioxide
UHC: Unburnt Hydrocarbons
W: Watt
W/m K: watts per metre-kelvin
%: Percentage
